# Patient-reported quality of life and adherence outcomes after integrating exclusive liquid meal replacement in patients with head and neck cancer undergoing chemoradiation: results from a phase II study

**DOI:** 10.3389/fonc.2024.1433503

**Published:** 2025-01-07

**Authors:** Luca F. Valle, Fang-I Chu, Xiaoyan Wang, Andrew Erman, Jackie Hernandez, Elizabeth Kaoh, Nicolas Edgar, Ann C. Raldow, Deborah J. Wong, Michael L. Steinberg, Amar U. Kishan, Robert K. Chin, John V. Hegde

**Affiliations:** ^1^ Department of Radiation Oncology, University of California, Los Angeles, Los Angeles, CA, United States; ^2^ Department of Medicine Statistical Core, University of California, Los Angeles, Los Angeles, CA, United States; ^3^ Department of Speech Pathology, University of California, Los Angeles, Los Angeles, CA, United States; ^4^ David Geffen School of Medicine, University of California, Los Angeles, Los Angeles, CA, United States; ^5^ Department of Medicine, Division of Hematology Oncology, University of California, Los Angeles, Los Angeles, CA, United States

**Keywords:** head and neck cancer, chemoradiation, nutrition, gastrostomy tube, quality of life

## Abstract

**Objectives:**

Preventing malnutrition during chemoradiation (CRT) for head and neck cancer is critical maximizing quality of life (QOL). We sought to assess patient-reported QOL outcomes after integrating exclusive liquid meal replacement with Soylent, a novel meal replacement agent, in patients with head and neck cancer undergoing CRT.

**Methods:**

Patients undergoing definitive or adjuvant concurrent CRT for locally advanced head and neck cancer enrolled on our single-institution, prospective phase II protocol evaluating nutritional replacement with Soylent. Patients who reached 5% body weight loss during CRT were transitioned to Soylent meal replacement for all nutritional needs. Patients who reached 10% body weight loss were recommended for gastrostomy tube (G-tube) placement. UW-QOL and FACT-H&N questionnaires assessed patient-reported QOL prior to the receipt of CRT and following conclusion of CRT. Paired t-test or Wilcoxon signed-rank test were performed to assess for differences between scores at each follow-up time point and baseline.

**Results:**

Of the 60 enrolled patients, 51/60 (85%) lost 5% of their pre-treatment body weight. Among these patients, 48/51 (94%) were successfully transitioned to Soylent. 22/48 patients subsequently lost 10% of their pre-treatment body weight, and 3/22 (14%) underwent G-tube placement with the remainder declining. This resulted in an overall G-tube rate of 5%. Among the 41 patients evaluable for QOL data, the nadirs for overall and health-related UW-QOL were reached at 1 month and rebounded to exceed baseline by 6 months. FACT-H&N survey scores were reduced from 32 at baseline to 20 at 1 month (adjusted p<0.001) and 26 at 3 months (adjusted p<0.001), but increased to 29, 30, and 27 at 6, 12, and 18 months, without significant differences as compared to baseline (adjusted p>0.38 for all).

**Conclusions:**

We report high patient adherence and a 5% G-tube placement rate with exclusive meal replacement with Soylent in patients undergoing concurrent CRT for head and neck cancers.

## Introduction

Concurrent chemoradiation (CRT) remains a curative, standard of care treatment for malignancies of the head and neck, both in the definitive and adjuvant setting. However, this treatment has been historically associated with significant short and long-term toxicities including mucositis, xerostomia, dysgeusia, dysphagia, nausea, and vomiting, and malnutrition (1).

Maintaining adequate nutrition to minimize weight loss during CRT for head and neck cancer is crucial for minimizing short and long-term treatment-related complications as well as maximizing treatment adherence, patient-reported quality of life (QOL), and cancer-related outcomes (2, 3). Oral nutritional supplementation is a common strategy for nutritional maintenance, though adherence can be challenging due to taste fatigue and treatment-related sequelae (4). If nutritional needs continue to be unmet, placement of a gastrostomy tube (G-tube) represents an invasive but often necessary escalation in the management of head and neck cancer patients undergoing CRT in order to ensure nutritional maintenance. While this strategy is reliable for enhancing nutrition, long-term swallowing dysfunction may increase when patients rely on G-tube feedings, leading to higher rates of permanent G-tube dependence (5, 6). Even in the modern treatment era, a recently published randomized de-escalation study of cetuximab vs cisplatin for human papilloma virus (HPV) positive oropharyngeal cancer (RTOG 1016) reported G-tube placement rates of 61.5% at treatment completion in the cisplatin arm which translated to a 9.2% G-tube placement rate at 1 year following treatment (7). Rates were nearly identical (57.3% and 8.4%) on the cetuximab arm. Strategies to maintain adequate nutrition during non-de-escalated CRT while simultaneously reducing G-tube dependence are thus desperately needed.

Soylent is a widely available meal replacement beverage that was designed to entirely fulfill human nutritional needs (8). It represents an attractive meal replacement solution for head and neck cancer patients owing to its taste fatigue-reducing formulation and comprehensive nutritional profile.

We sought to assess the compliance and QOL outcomes associated with complete nutritional replacement with Soylent in patients who experienced 5% weight loss during CRT. We hypothesized that meal replacement with Soylent would be well tolerated and would improve nutritional status, thereby reducing the historic rate of therapeutic G-tube insertion at our institution (30%) as well as the overall rate of G-tube placement on contemporary studies of head and neck cancer (61.5%).

## Methods

From August 2018 to March 2020, a total of 60 patients undergoing CRT for head and neck cancer were enrolled on a phase 2 single-institution trial of exclusive meal replacement with Soylent conducted at The University of California Los Angeles. Patients referred to radiation oncology for receipt of chemoradiation therapy were recruited to participate in the study by physicians in radiation oncology during their initial consultation visit.

Patients eligible for enrollment were required to have locally advanced head and neck malignancies for which CRT was recommended for definitive or adjuvant treatment. All radiotherapy was delivered using IMRT and conventional fractionation. Systemic therapy was delivered intravenously under the supervision of a medical oncologist. Patients were required to be ≥ 18 years of age with a Karnofsky Performance Status (KPS) ≥ 70, a body mass index (BMI) ≥18kg/m (2), without evidence of distant metastatic disease, and eligible to undergo concurrent chemotherapy as determined by the treating medical oncologist. Additionally, patients were not allowed to have gastrostomy tube (G-tube) prior to initiation of CRT, nor a history of prior radiotherapy to the head and neck. The CONSORT diagram can be found in **Supplementary Figure 1**.

Baseline weight was recorded on cycle 1, day 1 of chemotherapy. Once patients lost 5% of their baseline weight, they were recommended exclusive meal replacement with Soylent. For patients who subsequently lost 10% of their baseline weight, they were recommended G-tube placement. Crossing the 10% weight loss threshold within 3 days of projected treatment completion did not prompt a mandatory G-tube recommendation from the clinical team. Our co-primary endpoints were compliance with Soylent nutritional supplementation during concurrent CRT as well as the G-tube placement rate. Our secondary outcomes were patient-reported QOL scores.

### Patient-reported QOL assessments

QOL assessment was performed using 2 previously validated surveys administered at a pre-treatment baseline and at 1, 3, 6, 12, and 18 months of follow-up from completion of treatment. Patients were asked to complete these instruments in paper form in a private setting with the assistance of nursing staff if necessary.

The University of Washington Quality of Life scale (UW-QOL; version 4) is a survey used to evaluate patient-reported QOL outcomes in head and neck cancer (9). The UW-QOL consists of 12 domains pertaining to QOL in the categories of pain, appearance, activity, recreation, swallowing, chewing, speech, shoulder function, taste, saliva, mood, and anxiety. A score of 0 indicates very poor or no functional capacity with regard to that domain whereas a score of 100 indicates no disability in that domain. In the final part of the UW-QOL, patients were asked general questions focused on QOL. This segment was scored with 0 indicating very poor QOL and 100 indicating outstanding QOL, with a range of scores as integer values in between.

The Functional Assessment of Cancer Therapy-Head and Neck scale (FACT-H&N) is also a validated, multidimensional, self-reported QOL instrument specifically designed for use with patients with head and neck cancer (10). It consists of 27 core items that assess patient function in 4 domains (physical, social/family, emotional, and functional well-being), which is supplemented further by 12 site-specific items to assess for head and neck-related symptoms. Each item is rated on a Likert-type scale from 0 to 4, with higher scores representing better QOL, and then combined to produce subscale scores for each domain as well as a global QOL score. The FACT-General (FACT-G) subscore (encompassing physical, social, emotional, and functional well-being subscales) and FACT-H&N subscore (encompassing the head and neck-specific domain alone) were also calculated.

Any patient with a missing survey was excluded in the data set for that particular time point. Only patients with complete baseline pre-treatment questionnaires and at least one complete follow-up questionnaire were considered to have assessable quality of life survey data for the QOL subset analysis.

### Statistical analysis

QOL data were presented using descriptive statistics. With normality being assessed via quantile-quantile plot (Q-Q plot) and the Shapiro–Wilk test, paired t-test or the Wilcoxon signed rank test were employed, where appropriate, to compare QOL scores from baseline to follow-up time points. Adjusted p-values were also obtained via the Benjamini-Hochberg procedure to address the multiple testing problem with a false discovery rate (FDR) threshold of 0.2.

## Results

### Patient, tumor, and treatment characteristics

60 patients were enrolled. As presented in **Table 1**, the average age at enrollment was 58 years and the average KPS was 90. 72% of enrolled were male and the majority of patients identified as having non-Hispanic ethnicity (93%). 67% of patients had no history of smoking, whereas 15% had over a 30 pack-year history of smoking. Mean baseline weight was 181 lbs and mean baseline BMI was 27 kg/m2.

**Table 1:** Patient, tumor, and treatment characteristics.

Patient Characteristics	Number of Patients (%)
Sex	
Male	43 (72)
Female	17 (28)
Ethnicity	
Non-Hispanic	56 (93)
Hispanic	4 (7)
Smoking History	
None	40 (67)
<10 Pack Years	7 (12)
10-30 Pack Years	4 (7)
>30 Pack Years	9 (15)
Tumor Characteristics	Number of Patients (%)
Primary Tumor Site	
Oropharynx	27 (45)
Nasopharynx	9 (15)
Oral Cavity	7 (11.7)
Other	17 (29)
Cutaneous	6
Larynx	4
Paranasal Sinuses	3
Major Salivary Gland	1
Thyroid	1
Cavernous Sinus	1
Hypopharynx	1
AJCC 8th Edition T-Stage	
Tx	1 (2)
T1	10 (17)
T2	18 (30)
T3	13 (22)
T4	15 (25)
Recurrent	3 (5)
AJCC 8th Edition N-Stage	
Nx	1 (2)
N0	9 (15)
N1	22 (37)
N2	16 (27)
N3	3 (5)
Recurrent	3 (5)
AJCC 8th Edition Overall Stage Grouping	
x	1 (2)
I	10 (17)
II	14 (23)
III	15 (25)
IV	17 (28)
Recurrent	3 (5)
Treatment Characteristics	Number of Patients (%)
Total Radiation Dose	
70 Gy	43 (72)
66 Gy	9 (15)
60 Gy	8 (13)
Systemic Therapy	
Cisplatin	48 (80)
Carboplatin	6 (10)
Carboplatin/Taxol	3 (5)
Cetuximab	3 (5)
Treatment Setting	
Definitive	40 (67)
Adjuvant	20 (33)

The most common site treated was the oropharynx (27/60, 45%), followed by the nasopharynx (9/60, 15%). 96% (26/27) of all oropharynx cases were HPV positive, whereas 3/15 (20%) of nasopharyngeal cancers were HPV positive. Overall 8^th^ edition AJCC staging ranged from stage I (17%, 10/60) to stage IV (28%, 17/60).

The majority of patients were prescribed a total dose of 70 Gy (43/60, 72%), though patients were also treated to doses of 66 and 60 Gy. Most patients also received concurrent cisplatin (48/60, 80%), followed by carboplatin in 6/60 patients (10%).

### Weight loss outcomes, soylent adherence, and G-tube rates

**Figure 1:**
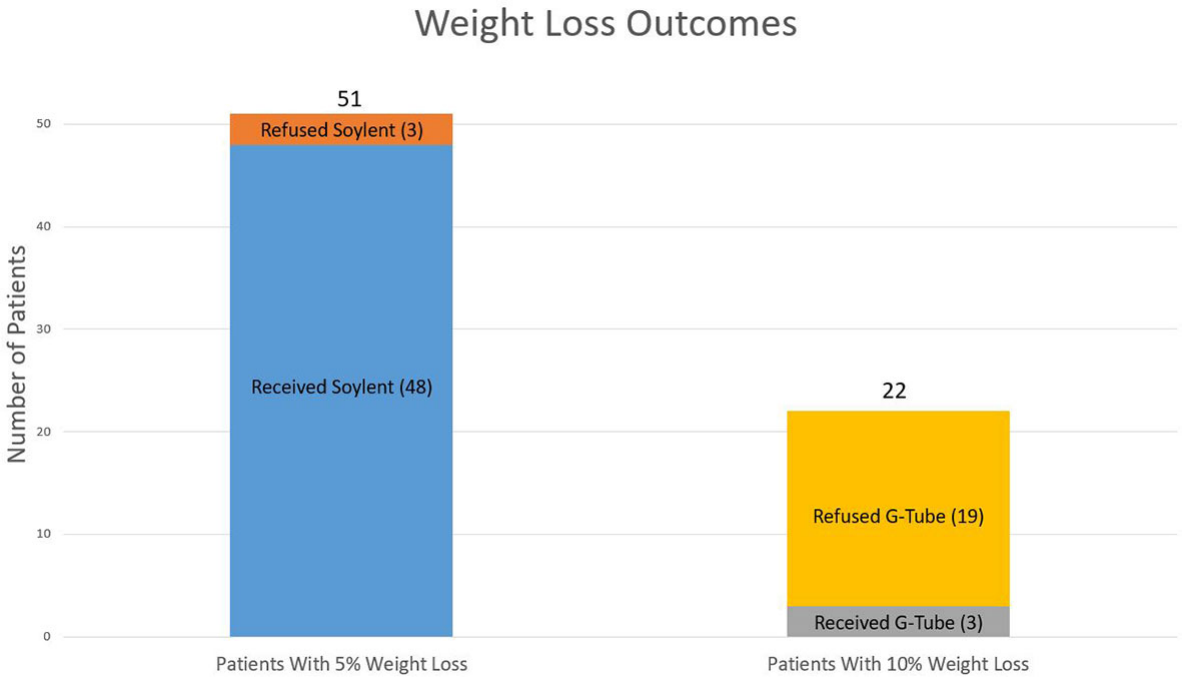
Weight loss outcomes. Weight loss outcomes from the 51 patients who lost 5% of their baseline weight as well as the 22 patients who lost 10% of their baseline weight.

51/60 (85%) enrolled patients lost 5% of their pre-treatment body weight. Among these 51 patients, all were offered exclusive meal replacement with Soylent, and 48/51 (94%) were successfully transitioned to Soylent. 3/51 (6%) patients either refused or did not tolerate full meal replacement with Soylent due to palatability concerns. Among the 48 patients who lost 5% of their body weight and were transitioned to Soylent, 22/48 patients lost an additional 5% of their pre-treatment body weight, prompting recommendation of G-tube placement. Ultimately, 3/22 (14%) actually underwent G-tube placement, whereas 19/22 (86%) declined G-tube placement (**Figure 1**). This translated to an overall G-tube rate of 5% (3/60) for the entire cohort.

### UW-QOL analysis

For both the UW-QOL and the FACT-H&N surveys, among the 41 patients with evaluable quality of life surveys, the baseline, 1-month, 3-month, 6-month, 12-month, and 18-month post-treatment UW-QOL survey completion rates were 100%, 83%, 73%, 45%, 20%, and 13%, respectively.

**Figure 2:**
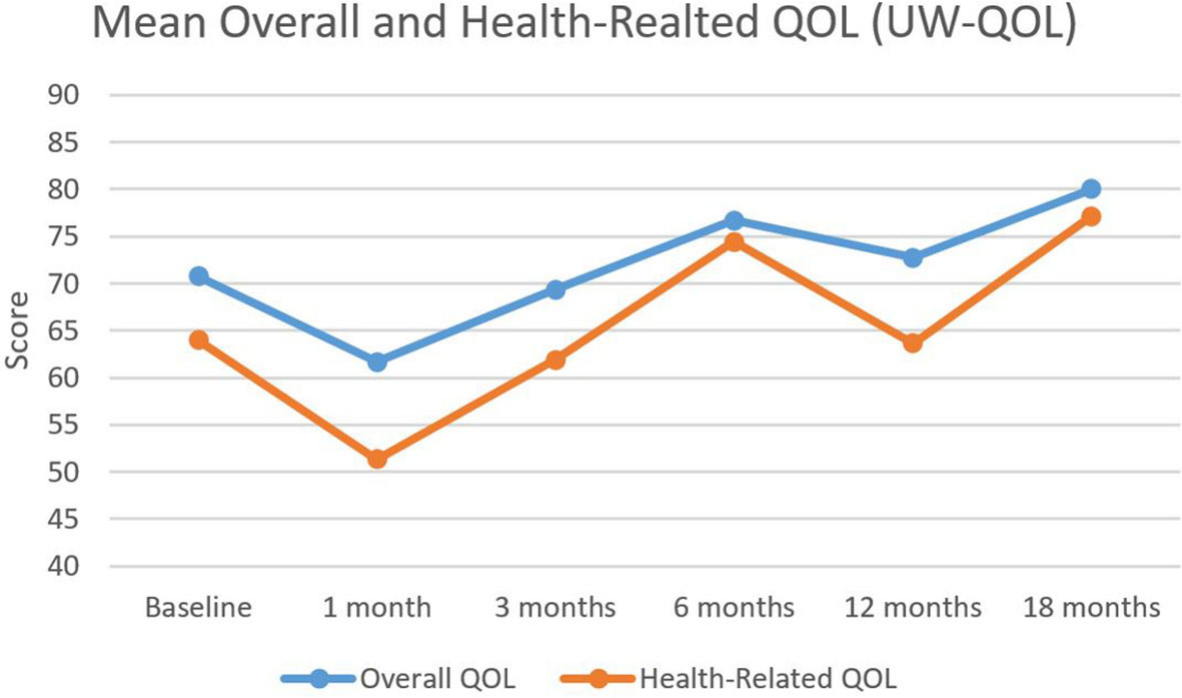
Mean overall and health-related QOL outcomes. Mean overall and health-related quality of life as assessed by the University of Washington Quality of Life (UW-QOL) survey instrument for the 41 patients with assessable questionnaires.

At baseline, the mean overall and health-related QOL scores as determined by the UW-QOL were 70.8 and 69.7, respectively. As illustrated in **Figure 2**, the nadirs for both measures were reached 1 month following completion of CRT, with overall QOL scores of 61.7 and health-related QOL scores of 51.3. Following the 1-month nadir, patients experienced an improvement in their overall and health-related QOL which numerically exceeded baseline by the 6-month time point, with a slight decline in scores at the 12-month mark, followed by a subsequent increase in scores to 80 and 77.1 by 18 months post completion of therapy. Overall QOL scores were not significantly different between baseline and any time point (**Table 2**).

**Table 2:** UW-QOL scores.

	Baseline	1 month	3 months	6 months	12 months	18 months
	(stdev)	(stdev)	(stdev)	(stdev)	(stdev)	(stdev)
**Overall Score**	70.8 (20.4)	61.7 (21.3)	69.4 (21.5)	76.7 (25.0)	72.7 (20.5)	80.0 (23.1)
Adjusted p-value	–	0.45	0.54	0.45	0.8	0.71
**Pain Subscore**	73.2 (25.9)	58.1 (27.3)	74.4 (26.2)	78.8 (21.9)	75.0 (24.0)	89.3 (19.7)
Adjusted p-value	–	0.35	1.00	0.82	0.82	1.00
**Appearance Subscore**	80.5 (20.5)	75.7 (15.7)	70.5 (18.2)	85.0 (18.9)	85.7 (12.8)	89.3 (13.4)
Adjusted p-value	–	0.61	**p=0.03**	0.27	1.00	0.75
**Activity Subscore**	76.8 (21.9)	55.9 (20.5)	71.2 (19.9)	73.8 (22.2)	71.4 (30.8)	82.1 (23.8)
Adjusted p-value	–	**p<0.01**	0.30	1.00	0.33	1.00
**Recreation Subscore**	78.1 (20.6)	58.1 (19.2)	70.5 (18.2)	82.5 (18.3)	75.0 (31.0)	89.3 (13.4)
Adjusted p-value	–	**p<0.001**	**p=0.02**	0.38	0.38	1.00
**Swallowing Subscore**	92.3 (15.8)	64.1 (26.8)	88.2 (14.9)	91.0 (14.1)	87.1 (15.4)	91.4 (14.6)
Adjusted p-value	–	**p<0.001**	**p=0.06**	0.48	0.22	0.43
**Chewing Subscore**	85.4 (23.05)	63.6 (33.7)	71.9 (28.2)	82.5 (24.5)	89.3 (21.3)	85.7 (24.4)
Adjusted p-value	–	**p<0.01**	**p=0.06**	0.77	0.77	0.77
**Speech Subscore**	91.2 (13.8)	83.8 (21.0)	85.5 (15.2)	94 (12.3)	91.4 (14.1)	91.4 (14.6)
Adjusted p-value	–	**p=0.10**	**p=0.10**	1.00	1.00	1.00
**Shoulder Subscore**	88.5 (21.3)	87.1 (30.1)	88.8 (26.4)	88.5 (27.2)	67.1 (27.6)	95.7 (11.3)
Adjusted p-value	–	1.00	1.00	1.00	**p=0.06**	1.00
**Saliva Subscore**	90.5 (18.5)	50 (28.2)	53.3 (29.3)	57.4 (32.6)	55.0 (29.8)	72.9 (23.6)
Adjusted p-value	–	**p<0.001**	**p<0.001**	**p<0.01**	**p<0.01**	**p=0.02**
**Taste Subscore**	89.3 (21.4)	25.8 (26.9)	63.6 (26.1)	67.5 (25.5)	67.1 (27.6)	90.0 (26.5)
Adjusted p-value	–	**p<0.001**	**p<0.001**	**p<0.01**	**p<0.01**	1.00
**Mood Subscore**	74.4 (21.0)	65.1 (25.3)	74.2 (22.1)	72.5 (21.3)	69.6 (28.0)	89.3 (19.7)
Adjusted p-value	–	0.21	0.94	1.00	0.47	1.00
**Anxiety Subscore**	51.2 (29.7)	67.7 (24.5)	62.7 (30.0)	78.0 (26.5)	64.3 (34.4)	81.4 (26.7)
Adjusted p-value	–	**p<0.01**	**p=0.06**	**p<0.01**	0.28	**0.11**
**Percent Change in Overall Score**	–	-12.85%	+18.36%	+20.62%	+24.15%	+32.06%

Table presents adjusted p-values. Bolded values are significant (p-value threshold of 0.2).

**Figure 3:**
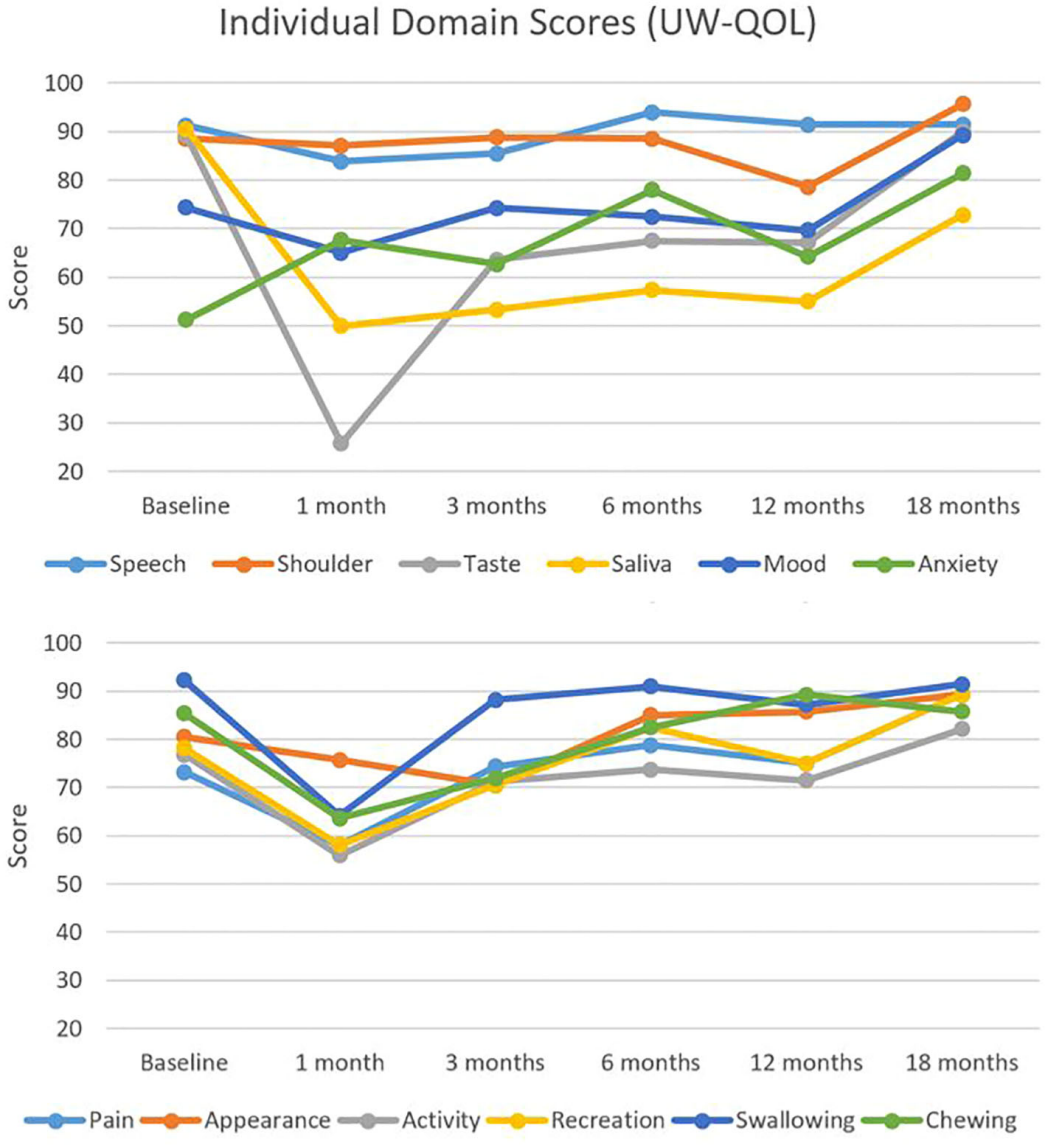
Individual domain quality of life scores. Individual head and neck domain scores as assessed by the University of Washington Quality of Life (UW-QOL) survey instrument for the 41 patients with assessable questionnaires.

**Figure 3** illustrates QOL scores segregated by domains relevant to the treatment of head and neck cancer. A similar trend was observed across most domains with a nadir at 1 month, which was especially prominent for taste. Scores eventually rebounded to exceed baseline by 18 months. Comparing individual domain scores from baseline to 18 months, significant differences were found in only two domains. Saliva scores decreased significantly from 91 to 73 (adjusted p-value = 0.02), whereas anxiety scores increased significantly from 51 to 81 (adjusted p-value = 0.11). Adjusted p-values for comparisons between all-time points and baseline are presented in **Table 2**.

### FACT H&N QOL analysis

The FACT-H&N QOL survey results are outlined in **Table 3**. At baseline, the mean total score, FACT-G subscore, and H&N subscore was 116.8, 85.2, and 31.6, respectively. At 1 month following completion of CRT, the corresponding values were 98.0, 78.3, and 19.7, respectively, each of which was significantly depressed from baseline and represented the nadirs during the post-treatment period (adjusted p<0.05 for all). However, at 3 months following treatment, only the total score and H&N subscore remained significantly depressed. By 6 months, all scores were not significantly different from baseline, and this remained the case until 18 months of follow-up (adjusted p>0.02 for all). The p-values for all FACT QOL comparisons can be found in **Table 3**.

**Table 3:** FACT-QOL scores.

	Baseline	1 month	3 months	6 months	12 months	18 months
	(stdev)	(stdev)	(stdev)	(stdev)	(stdev)	(stdev)
**Mean FACT-G Subscore (SD)**	85.2 (11.5)	78.3 (14.3)	83.8 (16.3)	85.4 (12.9)	87.9 (12.4)	93.5 (10.3)
Adjusted p-value	–	**p<0.02**	0.87	0.68	0.88	0.48
**Mean H&N Subscore (SD)**	31.6 (6.8)	19.7 (7.3)	26.0 (6.6)	28.8 (6.0)	29.7 (4.0)	26.7 (6.9)
Adjusted p-value	–	**p<0.001**	**p<0.001**	0.57	0.61	0.57
**Mean Total Score (SD)**	116.8 (16.0)	98.0 (19.9)	109.8 (21.4)	114.3 (17.5)	117.6 (15.4)	120.3 (16.1)
Adjusted p-value	–	**p<0.001**	**p<0.02**	1.00	1.00	1.00
**Percent Change in Total Score**	–	-16.06%	-5.98%	-2.11%	+0.69%	+02.96%

Table presents adjusted p-values. Bolded values are significant (p-value threshold of 0.2).

## Discussion

In patients undergoing conventional, non-deescalated concurrent CRT for head and neck cancer, we report a high adherence rate of 94% with exclusive Soylent meal replacement when patients lose 5% of their pre-treatment body weight. We also observed a 5% incidence rate of G-tube placement for the total cohort of enrolled patients, which compares favorably to our historical institutional G-tube placement rate of 30% and the G-tube rate of 61% reported on contemporary NRG studies (7). We also report a return to baseline patient-reported QOL according to two survey instruments by 6 months after a nadir in quality of life at 1 month post-treatment.

While several prospective studies have evaluated the value of oral supplementation during radiation alone for head and neck cancer, few studies have evaluated this in the setting of concurrent CRT, where side effects are more severe (11). These studies were also largely conducted in an era prior to modern intensity modulated radiotherapy (IMRT) for head and neck cancer, which has improved short-term and long-term toxicities like xerostomia and dysphagia, which can affect nutritional intake (12, 13). Our results suggest that even in the IMRT era, aggressive nutritional monitoring and oral liquid meal replacement contribute to the expeditious return of QOL and yield low G-tube rates.

The favorable oncologic outcomes for patients with head and neck cancers, especially in the HPV+ subset (14), has consequently refocused much needed attention onto strategies that improve QOL in this cancer population. In the modern treatment era, our study demonstrates favorable QOL outcomes are achievable following exclusive Soylent meal replacement and prompts reflection on the role of simple, creative, and low-tech avenues for improving QOL in head and neck cancer patients.

An interesting finding is the decrement in both overall and health-related QOL at 12 months as assessed by the UW instrument, in spite of prior gains in these metrics at the 3- and 6-month time points. This appeared to be driven by decrements in scores related to anxiety, shoulder motion, and recreation, which might be explained by anxiety surrounding surveillance imaging at the 1-year time point or late-developing fibrotic sequelae of CRT.

Though differences in patient populations and treatment techniques mean that direct comparisons with other studies evaluating separate research questions should be undertaken with caution, it is nonetheless interesting to appreciate the similarities in QOL profiles across similar time points in patients who were also enrolled on a phase II study of de-escalated CRT at our institution between October 2012 and March 2015. In a companion QOL analysis from patients enrolled on that trial, Hegde et al. also demonstrated return to baseline FACT-G subscores at 3 months, and normalization of FACT-HN subscores and mean total scores by 6 months. These findings represented an improvement in QOL outcomes with de-escalation compared to historical controls in their study and highlighted the promise of de-escalation efforts for improving QOL. That we observed similar time to baseline QOL recovery across similar metrics using identical survey instruments at the same institution suggests that aggressive nutritional replacement may plays a similarly powerful role in abrogating CRT toxicity in the treatment of head and neck cancers. In contrast however to the companion QOL study, patients in the present study did not experience a significant increase in FACT scores above baseline by 18 months, which is contextualized by the fact that these patients were receiving standard non-de-escalated CRT. It may also be worth noting that the G-tube rate on this study compares favorably with our institution’s historical G-tube rate of 30%.

An important limitation of this study is that we are not able to disentangle potential synergistic QOL effects between aggressive nutritional counseling, dietary monitoring, and consumption of Soylent as a meal replacement itself. However, Soylent as a nutritional agent is the subject of ongoing clinical investigation, including its impact on human microbiome composition (NCT 03203044). Thus, future studies may be able to better elucidate the relative benefits and tradeoffs of Soylent specifically as a nutritional agent in clinical settings.

It is also true that our favorable quality of life outcomes and low G-tube rates could be explained by a patient population with generally favorable performance status attributes or by improvements in supportive care, systemic therapies, and radiation therapy. However, even in modern series, G-tube rates can be quite high in patients receiving full dose standard of care CRT. For example, despite improvements in the delivery of local and systemic therapies, a recent multi-institutional study of patients with oropharyngeal cancer revealed that 82% of patients receiving concurrent CRT with IMRT required G-tube insertion, albeit approximately half in the therapeutic, rather than prophylactic, setting (15). However, we recognize that variation in institutional practice patterns is also likely to play a significant role in the rates of G-tube insertion. Specifically, patients undergoing CRT who engage in regular swallowing exercises alongside rigorous oral intake strategies have been shown to experience less severe treatment-related dysphagia (16), and thus rigorous referral of all patients undergoing head and neck CRT to speech language pathologists prior to treatment initiation (as is customary at our institution) may also simultaneously be driving the low G-tube rates reported herein.

Of course, treatment de-escalation is another approach for improving QOL that has become of significant interest in recent years, particularly for the HPV positive subset of head and neck cancer patients. Rates of feeding tube insertion from landmark studies (17) of de-escalation therapy do appear to be on-par with our rates, with pre-and post-treatment rates of 1.3% 2.8% in the 60 Gy plus cisplatin arm of NRG HN002 and rates of 0% and 3.8% in the 60 Gy alone arm. Moreover, in a recently published quality of life analysis from the single institution phase II de-escalation study MC1273, FACT-HN scores at 12 months were remarkably high at 117.2 pre-RT, which increased to 127.2 at 12 months of follow-up (18). While this study focused exclusively on patients requiring adjuvant therapy and thus only a subset of patients received CRT as part of trimodality therapy, this nonetheless suggests that deploying treatment de-escalation strategies may result in patient-reported QOL gains that surpass nutritional interventions alone, but are likely to be complementary to QOL gains achieved from nutritional intervention. However, it is important to note that at the time of this writing, the safety of de-escalation has yet to be proven in the randomized phase 3 setting, and thus standard of care for all head and neck cancer patients remains full dose CRT. Aggressive nutritional management with nutritional adjuncts such as Soylent may serve as an effective bridge to maintaining high quality of life even in the face of full dose CRT while the results of practice-changing de-escalation studies are eagerly awaited.

Maintenance of QOL during treatment for head and neck cancer results from a complex interplay of patient, tumor, treatment, and provider-related factors. However, predictors of poor QOL and thus appropriate mitigating strategies are sometimes unexpected, as illustrated by a recent narrative literature review suggesting that patients undergoing curative intent protocols who were more advanced in age tended to demonstrate increased resilience and QOL outcomes (19) when compared to their younger counterparts. Thus, if our prevailing aim is to reduce toxicity from curative head and neck CRT courses, our efforts should not simply focus on the “high-tech” intuitive strategies such as reducing radiation or cytotoxic chemotherapy dosing, but instead should leverage the full spectrum of modern multidisciplinary cancer care (including “low-tech” solutions such as aggressive nutritional monitoring and supplementation) in order to make meaningful inroads in the improvement of QOL outcomes for this critical population of cancer patients.

## Data Availability

The raw data supporting the conclusions of this article will be made available by the authors, without undue reservation.
