# Supplementary figures and images for: Patient-reported quality of life and adherence outcomes after integrating exclusive liquid meal replacement in patients with head and neck cancer undergoing chemoradiation: results from a phase II study
Source: Front Oncol. 2025 Jan 7;14:1433503. doi: 10.3389/fonc.2024.1433503 (PMC11747389; doi:10.3389/fonc.2024.1433503)

**Supplemental Figure 1: CONSORT Diagram**

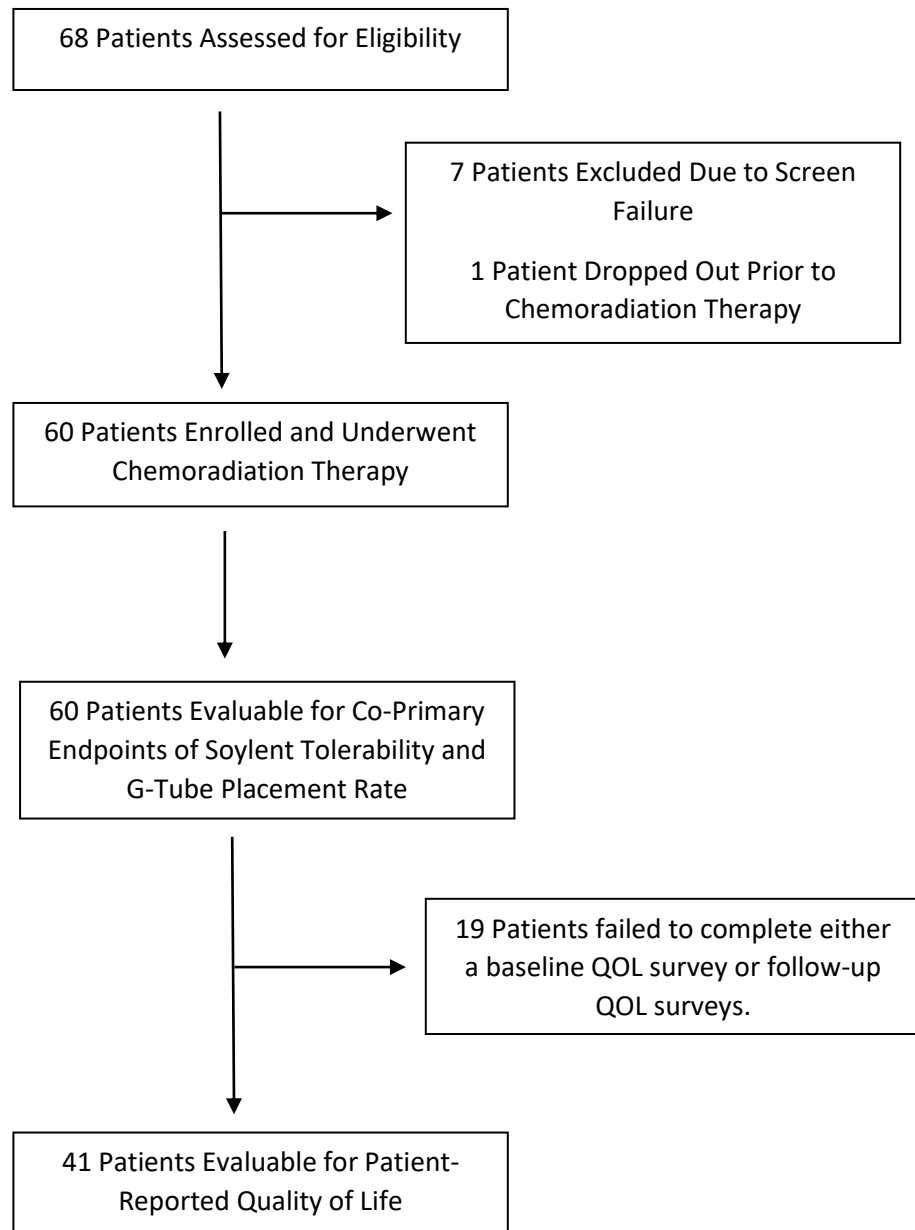

Supplement: Supplementary file 1 [file DataSheet1.pdf]
