# Supplementary material for: Patient-reported quality of life and adherence outcomes after integrating exclusive liquid meal replacement in patients with head and neck cancer undergoing chemoradiation: results from a phase II study
Source: Front Oncol. 2025 Jan 7;14:1433503. doi: 10.3389/fonc.2024.1433503 (PMC11747389; doi:10.3389/fonc.2024.1433503)
Supplement: Supplementary file 2 [file DataSheet2.pdf]

A Phase II Study Integrating Soylent^{TM} Meal Replacement to Reduce Gastrostomy Tube Rates in Patients with Head & Neck Cancer Undergoing Chemoradiotherapy

Principal Investigator:
John V. Hegde, M.D.
PGY-4, UCLA Radiation Oncology

Faculty Mentor:
Robert K. Chin, M.D., Ph.D.
Assistant Professor, UCLA Radiation Oncology

Co-Investigators:
Robert K. Chin, M.D., Ph.D.
Assistant Professor, UCLA Radiation Oncology

Andrew A. Erman, MA/CCC-SLP
Clinic Director, UCLA Speech Pathology Clinic

Ann Raldow, MD, MPH
Assistant Professor, UCLA Radiation Oncology

Joanne B. Weidhaas, MD, PhD, MSM
Professor, UCLA Radiation Oncology

Dörthe Schaue, MRes, Ph.D.
Associate Professor, UCLA Radiation Oncology

Deborah J. Wong, M.D., Ph.D.
Assistant Professor, Hematology-Oncology, UCLA Medicine

Michael L. Steinberg, M.D.
Professor and Chair, UCLA Radiation Oncology

Amar Kishan, M.D.
Assistant Professor, UCLA Radiation Oncology

Albert J. Chang, M.D., Ph.D.
Assistant Professor, UCLA Radiation Oncology

Victoria A. Ramirez UCLA Study Coordinator

Brian Carlton Regulatory Specialist

Elizabeth Koah, Registered Dietitian
Hoda Hakimjavadi, Registered Dietitian

Study Center:
UCLA Jonsson Comprehensive Cancer Center
Department of Radiation Oncology

200 UCLA Medical Plaza, Suite B265
Los Angeles, CA 90095

INDEX

PROTOCOL SYNOPOSIS

SCHEMA

1. **OBJECTIVES**
2. **BACKGROUND**
3. **PATIENT SELECTION**
4. **REGISTRATION PROCESS**
5. **TREATMENT PLAN**
6. **PHARMACEUTICAL INFORMATION**
7. **ADVERSE EVENTS: LIST AND REPORTING REQUIREMENTS**
8. **STUDY CALENDAR**
9. **DATA REPORTING/REGULATORY CONSIDERATIONS**
10. **STATISTICAL CONSIDERATIONS**
11. **APPENDIX**
12. **REFERENCES**

PROTOCOL SYNOPOSIS:

CONCEPT/RATIONALE:

Maintaining adequate nutrition to minimize weight loss during chemoradiotherapy for head and neck cancer is crucial for treatment adherence, minimizing short- and long-term treatment-related complications, quality of life (QOL), and cancer-related outcomes[1-6]. However, as chemoradiation may result significant acute toxicities including mucositis, xerostomia, Dysgeusia, dysphagia, nausea, and vomiting, the rate of malnutrition in these patients reaches 44-88% during treatment[7].

Several methods to improve nutritional status are commonly employed during chemoradiation. One common intervention is oral nutritional supplementation with commercially-available calorie-dense shakes like Boost® and Ensure®. Importantly, compliance with oral nutritional supplementation is challenging due to taste fatigue and treatment-related sequelae[8]. Another commonly used intervention is the routine use of nutritional counseling, which has been shown, with or without oral nutritional supplementation, to have a positive impact during radiation and have a durable impact on QOL[9-11]. Lastly, gastrostomy tube (G-tube) placement for enteral feedings is a third, and invasive, method for nutritional maintenance. While reliable for enhancing nutrition, long-term swallowing dysfunction may increase when patients rely on G-tube feedings for nutrition during chemoradiation, leading to higher rates of permanent G-tube dependence[12-14].

While several prospective studies have evaluated the value of oral supplementation during radiation alone for head and neck cancer, few studies evaluate this in the setting of concurrent *chemo*radiation, where side effects are more severe[15]. These studies were also largely conducted in an era prior to modern intensity modulated radiotherapy (IMRT) for head and neck cancer, which has improved short-term and long-term toxicities like xerostomia and dysphagia, which can affect nutritional intake[16-18]. Even with these improvements in radiotherapy delivery, a recent multi-institutional study of patients with oropharyngeal cancer revealed that 82% of patients receiving concurrent chemoradiation with IMRT had a G-tube inserted (about half in the therapeutic, rather than prophylactic, setting)[19]. Thus, there is ample opportunity to improve and evaluate the utility of structured oral supplementation with IMRT in patients with locally advanced disease necessitating chemoradiation.

Soylent™ is a novel oral nutritional supplement designed to be a complete meal replacement agent that is widely available to the general public, including through Amazon.com. In contrast to more commonly available oral supplements, it contains no sugar and is formulated to reduce taste fatigue, which may improve compliance in patients with head and neck cancer. Therefore, we hypothesize that complete nutritional replacement by Soylent™ triggered by 5% weight loss may be able to minimize further weight loss and improve nutritional status so as to reduce the therapeutic G-tube rate as compared to standard chemoradiation and supportive care at our institution. G-tube insertion will be recommended at 10% weight loss. Improved nutritional status from Soylent™ supplementation may improve toxicity, QOL, clinical outcomes, and may be associated with biomarkers predictive of treatment response or prognostic for cancer-related outcomes.

PRIMARY OBJECTIVE (S):

(1) To determine the compliance rate of Soylent™ oral nutritional replacement
(2) To determine the therapeutic G-tube placement rate (G-tube placement from the 1st day of chemoradiation up to 4 weeks following treatment)

SECONDARY OBJECTIVE (S):

(1) To determine weight loss and body mass index (BMI) changes during and following treatment
(2) To determine the change in nutritional and metabolic biomarkers during treatment
(3) To determine physician-reported acute and late toxicities during and following treatment
(4) To determine patient-reported quality of life during and following treatment
(5) To determine clinical outcomes from treatment

PRIMARY ENDPOINT (S):

(1) Compliance rate of oral nutritional supplementation
(2) Therapeutic G-tube placement rate

SECONDARY ENDPOINT (S):

(1) Weight loss
(2) Body mass index (BMI)
(3) Treatment breaks
(4) Timing and duration of G-tube placement
(5) Serum HPV DNA, pre-albumin, albumin, C-reactive protein (CRP), interleukin-6 (IL-6), tumor necrosis factor-α (TNF- α), Vitamin A, Vitamin C, Vitamin D, tryptophan, kynurenine, leptin, and adiponectin, complete metabolic panel, and complete blood count
(6) Radiosensitivity germ-line biomarker panel
(7) Physician-reported acute and late toxicities according to the CTCAE version 4.0
(8) Patient-reported quality of life according to the University of Washington and Functional Assessment of Cancer Therapy questionnaires
(9) Local/Locoregional control
(10) Distant metastasis-free survival
(11) Overall survival

STUDY DESIGN:

Single arm, phase II prospective study to evaluate Soylent™ oral nutritional supplementation for compliance rate and impact on therapeutic G-tube rate, as well as its potential effect on nutrition, quality of life, and clinical outcomes with its use during chemoradiation in the definitive or adjuvant setting for head and neck cancer.

NUMBER OF PATIENTS:

60

ELIGIBILITY CRITERIA:

- Documented locally advanced head and neck malignancies for which concurrent chemoradiation has been recommended for definitive or adjuvant treatment

No history of prior radiotherapy to the head and neck; however, if previous treatment was superficial skin radiotherapy without regional lymph node treatment this will be allowed.

- Age ≥ 18 years
- Karnofsky Performance Status (KPS) ≥ 70
- Body mass index ≥18kg/m^2
- No evidence of distant metastatic disease (M1 disease)
- No G-tube placement prior to initiation of chemoradiation
- Eligible to undergo concurrent chemotherapy as determined by treating medical oncologist
- If a woman is of childbearing potential, a negative urine pregnancy test must be documented prior to proceeding with chemoradiation. Women of childbearing potential must agree to use adequate contraception (hormonal or barrier method of birth control; or abstinence) for at least 4 weeks after study treatment
- Ability to understand and willingness to sign a written informed consent
- Able to tolerate the taste of the oral nutritional supplement Soylent™ at the time of screening.
- Able and willing to participate in the Swallow Preservation Program at the Speech Pathology Clinic
- No evidence of clinically significant swallowing dysfunction by history or physical exam at time of radiation oncology consultation

INTERVENTION AND MODE OF DELIVERY:

Intervention is a commercially-available oral nutrition supplement. It will be provided by the study team to patients who are enrolled when their weight loss exceeds 5% from baseline. Patients will drink the supplements for nutrition during their meal times as directed by their clinical dietitian during and up to 1 month following chemoradiation. Patients will be recommended for Soylent™ supplementation as their only means of nutrition during the intervention period.

DURATION OF INTERVENTION AND EVALUATION:

Nutritional supplementation will take place if weight loss exceeds 5% from the baseline weight taken at study enrollment during concurrent chemoradiation. This is expected to occur in approximately 90% of patients undergoing chemoradiation from our institutional historical control data. Nutritional supplementation may continue until the month following completion of chemoradiation (up to approximately 3 month's total). Patients will be evaluated weekly by the study site's clinical dietitian and clinical staff to assess weight changes as is done for all patients undergoing chemoradiation to the head and neck. Patients will remove their shoes, items from their pockets, and any coats or sweaters. The dietitian and study staff will also determine the amount of supplementation utilized and assess the need of a G-tube during chemoradiation. G-tube placement will be recommended if a patient reaches 10% weight loss and/or is not meeting nutritional requirements as assessed by a dietitian. Patients will be required to be in a swallow preservation program with the Speech Pathology Clinic for minimizing dysphagia and aspiration surveillance. Clinical nutritional follow-up will continue as long as the patient is recommended to have nutritional supplementation. Weight measurements will be routinely taken at every follow-up visit. Blood/cheek swab sample collection (whenever feasible) for future analysis may occur at baseline (prior to treatment), at the conclusion of treatment (last week of chemoradiation), and at the 3-month follow-up visit. The University of Washington Quality of Life (UW-QOL) and Functional Assessment of Cancer Therapy Head & Neck (FACT-HN) questionnaires will be used

to assess QOL prior to beginning chemoradiation and at every routine follow-up after completing chemoradiation. Routine clinical follow-up will last for a minimum of 2 years.

STATISTICAL METHODS:

SAMPLE SIZE JUSTIFICATION:
Phase II: This study is designed to examine compliance and efficacy. That is, whether patients who require oral supplementation with Soylent™ continue using the supplement during treatment as directed ($\geq$ 50% of the time), and whether therapeutic G-tube rates are reduced by 33% with the utilization of oral nutritional supplementation with Soylent™ compared to our modern cohort of institutional historical control patients (treated from 2015-2016) who had received chemoradiation for head and neck cancer with standard nutritional counseling at our institution. One primary endpoint is to ensure compliance with Soylent™ nutritional supplementation during concurrent chemoradiation. For feasibility of using this meal replacement, we set a goal of more than 50% of patients should continue 50% of recommended daily Soylent intake. A therapeutic G-tube placement rate 33% lower than institutional historical control data is the second primary endpoint for this study. Our historical control data notes a 32% G-tube rate for chemoradiation patients, and we hypothesize reduction in g-tube placement to 20% of our patient population. If, conversely, a 25% higher rate of therapeutic G-tube placement occurs (equating to a 40% G-tube placement rate), study stoppage at an interim analysis period following treatment of 30 patients will be triggered. A sample size of 60 patients achieves 80% power to detect a 33% reduction in G-tube placement rate when comparing to historical control patients (i.e. 20% vs 32%), using a one-sided one-sample exact Binomial test, at a 0.10 significance level.

FUNDING, REGULATORY, AND FEASIBILITY ISSUES:
The UCLA Department of Radiation Oncology has the capability, equipment, and expertise to provide oral supplementation, evaluate nutritional status, weight changes, and the need for a therapeutic G-tube during treatment. Clinical and radiographic follow-up will be performed per standard of care with blood samples and questionnaires issued per study protocol. Study associated costs outside of standard of care will be covered by the Department of Radiation Oncology.

PATIENT ACCEPTABILITY/ETHICS AND CONSENT ISSUES:
Only patients able to give informed consent will be eligible for the study.

SCHEMA

Patient meets with radiation oncologist at consultation or follow-up visit, who has recommended chemoradiation in the definitive or adjuvant setting for head and neck malignancies

↓

Patient eligibility determined by study staff

↓

Patient agrees to enroll in study
Baseline serum and cheek swab sampling, QOL

↓

Clinical nutrition and speech pathology assessments

↓

Initiation of Soylent™ nutritional replacement with 5% weight loss from baseline weight taken prior to treatment. Replacement may continue for up to 3 months total (2 months during chemoradiation and 1 month after)

↓

Weekly evaluation by clinical

Dietitian and study staff recording weight changes, oral intake, compliance with Soylent™ , need for a therapeutic G-tube (i.e. >10% weight loss)

↓

Continued routine clinical follow-up for 2 years, including weight, clinical outcomes, QOL

1.0 OBJECTIVES

1.1 Primary objectives

1.1.1 To determine the compliance rate of oral nutritional replacement with Soylent™
1.1.2 To determine the overall therapeutic G-tube placement rate (G-tube placement from the 1st day of chemoradiation up to 4 weeks following treatment)

1.2 Secondary objectives

1.2.1 To determine weight loss and body mass index (BMI) changes during and following treatment
1.2.2 To determine the change in nutritional and metabolic biomarkers during and following treatment
1.2.3 To determine physician-reported acute and late toxicities during and following treatment
1.2.4 To determine patient-reported quality of life during and following treatment
1.2.5 To determine clinical outcomes from treatment

2.0 BACKGROUND

Maintaining adequate nutrition to minimize weight loss during concurrent chemoradiotherapy for head and neck cancer is important for treatment adherence, treatment-related complications, quality of life (QOL), and even potentially clinical outcomes like death[1-6]. However, chemoradiation usually results in multiple acute toxicities which make maintaining adequate nutritional intake challenging, including mucositis, xerostomia, dysgeusia, dysphagia, nausea, and vomiting. Therefore, avoiding malnutrition can be difficult, especially given that some patients (up to 52%) already have evidence of malnutrition prior to treatment commencing[7]. Once on treatment, up to 88% of patients may have evidence of malnutrition[7].

Several methods to improve nutritional status are commonly employed during chemoradiation. One common intervention is oral nutritional supplementation with commercially-available shakes like Boost® and Ensure®, often initiated by clinicians early in treatment. A study of 23 patients randomized to either radiation alone or radiation with oral supplementation noted that all patients receiving nutritional supplementation had an increase in body weight, while 58% of the radiation alone group had weight loss [20]. Additionally, no radiation treatment breaks were needed in the oral supplementation group, while 5/12 patients in the radiation alone group needed treatment breaks due to severe mucositis or poor performance status. Therefore, while a small study, oral supplementation appeared to benefit patients from a nutritional and toxicity perspective. Another prospective study of 50 patients treated by definitive radiation for head and neck cancer randomized patients to oral supplementation with Sustacal™ for 10 weeks or no supplementation [21]. Notably, in this study patients having concurrent chemotherapy were excluded. The results of this study showed that although total protein and total calorie intake were increased in the supplemental group, equal levels of weight loss occurred. Furthermore, no differences in treatment response, complications, or survival were seen. Notably, only 67% of men and 56% of women consumed the recommended volume of supplementation (which contained 960-1080 kcal/day). Indeed, compliance with oral nutritional supplementation is challenging, both due to taste fatigue and treatment-related side effects[8]. Even in other cancer sites, patient compliance with dietary interventions could be further optimized, with rates ranging from 47-69% in several studies[22,23].

Another commonly used intervention is the use of nutritional counseling, often in combination with oral nutritional supplementation. Nutritional counseling with or without oral supplementation has been shown to have a significant impact during (chemo) radiation and actually have a durable impact on QOL[9-11]. One prospective, single-arm study of 21 patients noted relatively low levels of acute chemoradiation toxicity (33.3% with grade 3 mucositis) and nutritional decline with nutritional counseling and oral nutritional supplementation[9]. A randomized study evaluating 75 head and neck cancer patients undergoing chemoradiation or radiation alone had 3 arms: dietary counseling with regular foods, usual diet with supplementation, and usual diet alone[10]. The results showed significantly better oral intake and reduced incidence/severity of acute toxicities with dietary counseling as compared to either the usual diet with supplementation or usual diet alone.

Gastrostomy tube (G-tube) placement for enteral feedings is a third, and most invasive, method for nutritional maintenance in patients. This intervention is often recommended due to a contraindication for oral feeding like a high aspiration risk or patient refusal of adequate oral intake. Early G-tube insertion if it is in the best medical judgement by the treating physicians may be done prior to the 10% weight loss.

Early G-tube placement has been associated with reductions in weight loss, hospitalization from nutritional deficits, and persistent weight loss following treatment[24]. While this is the most reliable way to enforce nutrition, long-term swallowing outcomes appear to suffer when patients do not swallow food during chemoradiation and instead rely upon G-tube feedings[12-14]. Of note, G-tube dependence has been loosely associated with decreased survival metrics[25]. Alternatively, nasogastric tube (NG-tube) feedings may promote a faster return to oral nutrition[13], but are generally not preferred given the significant patient discomfort and emotional distress associated with NG tubes. No survival differences have been shown by using prophylactic versus therapeutic (reactive) G-tube interventions to optimize nutritional status[15].

Important to note, many of the above randomized studies were performed without the use of concurrent chemotherapy. Also, these previous studies evaluating oral supplementation were largely conducted in an era prior to modern intensity modulated radiotherapy (IMRT) for head and neck cancer, which has improved short-term and long-term toxicities like xerostomia and dysphagia, which can in turn affect nutritional intake[16-18]. A recent multi-institutional study of patients receiving definitive IMRT for oropharyngeal cancer revealed 63% had a G-tube placed, with 48% having reactive (vs. prophylactic) G-tube placement[19]. Importantly, 82% of patients having concurrent chemoradiation had a G-tube inserted. Thus, there is an opportunity to evaluate the utility of oral supplementation with IMRT in patients with locally advanced disease necessitating chemoradiation. Additionally, patient-reported QOL has, to our knowledge, never been evaluated using an oral supplement intervention for chemoradiation, although it has been reported in patients receiving radiation alone[10].

Multiple studies have noted associations between metabolic and nutritional biomarkers with the risk of malignancy, treatment-related toxicities, and cancer-related outcomes. Serum albumin

level is a marker which correlates with mortality in head and neck cancer patients[26]. Pre-albumin is a more sensitive marker than albumin in head and neck cancer patients undergoing RT[27]. Chemoradiation has been associated with changes in BUN, creatinine, bicarbonate, magnesium, albumin, sodium, and potassium levels[28]. C-reactive protein appears to increase during chemoradiation[29]. Vitamins C and E appear to mitigate RT-induced xerostomia following treatment for head and neck cancer[30]. Mixed evidence has been reported regarding the association of vitamin D levels with head and neck cancer outcomes[31].

Targeted supplementation to increase clinically-relevant biomarkers have been explored in head and neck cancer. One small study utilized omega-3 fatty acid and arginine-enhanced supplementation in surgical head and neck cancer patients, which resulted in improved blood protein concentrations, lymphocyte levels, and weight[32]. Another study with arginine-enhanced supplementation in post-operative head and neck cancer patients showed significantly decreased fistula formation[33,34]. In a third small study where patients with head and neck cancer underwent chemoradiation or palliative chemotherapy, patients randomized to a supplement regimen with added omega-3 fatty acids, micronutrients, and probiotics versus a control supplement[35]. This showed significantly improved weight, higher serum albumin, and higher pre-albumin.

Molecular biomarkers may also be helpful in predicting treatment response and prognosis in head and neck cancers. HPV is known to predict treatment response and prognosis in HPV-associated head and neck cancers of the oropharynx[36-38]. In head and neck cancers of the oral cavity, saliva is preferentially enriched for tumor DNA, while for other subsites plasma DNA is preferentially enriched[39]. Associating nutritional status to HPV DNA levels may be revealing.

Soylent™ is a novel, widely available, oral nutritional supplement designed to be a complete meal replacement agent. In contrast to commonly utilized oral supplement preparations, it does not rely on sugar to provide caloric intake, and it has a taste designed to reduce taste fatigue, which may improve compliance. Therefore, we hypothesize that Soylent™ would have a compliance rate which would make its use feasible for patients undergoing head and neck cancer chemoradiation. Also, we hypothesize that complete meal replacement starting at 5% weight loss, rather than ad hoc supplementation, may be able to minimize weight loss, improve nutritional status, and reduce the therapeutic G-tube rate significantly better than traditional methods in a modern cohort of head and neck cancer patients treated with chemoradiation. Furthermore, Soylent™ supplementation may affect nutritional, metabolic, and molecular biomarkers predictive for treatment response or for cancer-related outcomes. Lastly, we hypothesize that Soylent™ supplementation may impact toxicity, QOL, and cancer-related outcomes.

3.0 PATIENT SELECTION

3.1 Conditions for patient eligibility

3.1.1 Documented locally advanced head and neck malignancies for which concurrent chemoradiation has been recommended for definitive or adjuvant treatment

3.1.2 No history of prior radiotherapy to the head and neck. However, if previous treatment was superficial skin radiotherapy without regional lymph node treatment this will be allowed.

3.1.2.1

- 3.1.3 Age ≥ 18 years
- 3.1.4 Karnofsky Performance Status (KPS) $\geq$ 70
- 3.1.5 Body mass index $\geq$18 kg/m^2
- 3.1.6 No evidence of distant metastatic disease (M1 disease)
- 3.1.7 No G-tube placement prior to initiation of chemoradiation
- 3.1.8 Eligible to undergo concurrent chemotherapy as determined by treating medical oncologist
- 3.1.9 If a woman is of childbearing potential, a negative urine pregnancy test must be documented prior to proceeding with chemoradiation. Women of childbearing potential must agree to use adequate contraception (hormonal or barrier method of birth control; or abstinence) for at least 4 weeks after study treatment.
- 3.1.10 Ability to understand and willingness to sign a written informed consent
- 3.1.11 Able to tolerate the taste of one of the flavors of the oral nutritional supplement Soylent™
- 3.1.12 Able and willing to participate in the Swallow Preservation Program at the Speech Pathology Clinic
- 3.1.13 No evidence of clinically significant swallowing dysfunction by history or physical exam at time of radiation oncology consultation

3.2 Conditions for patient ineligibility

3.2.1 Patients who have previously received therapeutic radiation therapy to the head and neck except for superficial skin radiotherapy is allowed.

3.2.2 Patients who had G-tube placement due to concern for aspiration or due to severe malnutrition in advance of chemoradiation

3.2.3 Patients underweight in advance of chemoradiation (BMI < 18 kg/m^2)

3.2.4 Patients who refuse to use Soylent™ oral nutritional supplementation due to its taste or other patient preference reasons

3.2.5 Patients with allergies to any of the ingredients contained in the nutritional supplement

3.2.6 Pregnant women, or women of childbearing potential who are sexually active and not willing/able to use medically acceptable forms of contraception for the entire study period and for up to 4 weeks after the study treatment.

3.2.7 Refusal to sign the informed consent

3.2.8 Refusal to participate in the Swallow Preservation Program prior to start of Soylent if applicable.

3.2.9 Evidence of clinically significant swallowing dysfunction by history or physical exam at time of radiation oncology consultation

3.2.10 Distant metastatic disease (M1 disease)

4.0 REGISTRATION PROCEDURES

4.1 General guidelines

Patients seen at UCLA in the Department of Radiation Oncology and recommended for definitive or adjuvant chemoradiation for locally advanced head and neck cancer will be informed of this clinical trial if eligible. The decision to participate will be voluntary. Eligible patients who decides not to participate will continue to be offered standard chemoradiation therapy with standard supportive care (including nutritional counseling, oral nutritional supplementation with commercially available supplements, and/or therapeutic G-tube placement at 10% weight loss).

4.2 Registration Process

Informed consent form will be given to the patient for review. Consent will be obtained after a clear and thorough discussion between the patient and the study investigator in clinic. When practical and in the best interest of the subject, the consent discussion may occur by telephone/telemedicine. The IRB-approved informed consent document may be sent to the subject electronically to be signed and returned electronically to the investigator for counter-signature. To register a patient, the research coordinator will obtain or complete: (1) documentation of locally advanced head and neck cancer for which chemoradiation has been recommended (2) successful taste test of Soylent^{TM} nutritional supplement (3) signed informed consent form; (4) signed HIPAA authorization form.

Prior to confirmation of eligibility and enrollment in the study, the following will be obtained anytime in advance of beginning chemoradiation: (1) Total amount of blood samples that can potentially be collected 82.5 mL (27.5 mL at each draw), (2) cheek swabs sample, (3) quality of life assessments.

5.0 TREATMENT PLAN

5.1 Radiation Simulation and Planning

Enrolled patients, after confirmation of eligibility, will undergo standard radiation simulation and treatment planning for their head and neck cancer. Intensity-modulated radiation therapy (IMRT) techniques will be required for enrollment on this study.

The responsible study investigator(s), will delineate the regions of gross tumor volume (GTV) using all available imaging. Clinical target volume(s) (CTVs) will be delineated to account for regions at risk for harboring subclinical disease. Planning target volume(s) (PTVs) will be delineated by expansions to the CTV(s) to account for patient set-up error and motion.

Delineation of normal critical structures of the head and neck will be performed, including the brain, brainstem, spinal cord, eyes, lens, optic chiasm, optic nerves, oral cavity, lips, mandible, salivary glands, pharynx, esophagus, larynx, and cochlea. The radiation physicist will optimize the radiation therapy treatment plan and the responsible study investigator(s) will review it prior to approval for treatment. Dose volume histograms (DVH), and normal tissue constraint parameters specified below will be used to judge the quality of the plan and optimize doses to the PTV(s) as well as maximally sparing of organs at risk (OARs).

Treatment will be delivered in 30-35 daily sessions (Monday-Friday) lasting 15-30 minutes in the Department of Radiation Oncology.

5.2 Chemotherapy

Enrolled patients, after confirmation of eligibility, will undergo concurrent chemotherapy according to the prescription of their medical oncologist. Chemotherapy regimens may include weekly cisplatin (50 mg/m^2), bolus q3week cisplatin (100 mg/m^2), or weekly cetuximab (400 mg/m^2 loading dose with 250 mg/m^2 weekly infusion), although other chemotherapy regimens are permitted by medical oncologist preference. Infusions will occur in the hematology/oncology infusion center, and serum chemistries and complete blood count will be taken and reviewed per standard protocol during chemotherapy.

5.3 Baseline Nutritional, Metabolic, & QOL Assessments

Prior to beginning chemoradiation, baseline serum samples, cheek swab samples, body weight, and body mass index will be obtained. This may be collected at the time of consent. A panel of germ-line biomarkers predicting radiation-related toxicity will be incorporated to control for baseline differences in radiosensitivity. Baseline labs will be drawn along with necessary pre-treatment labs for chemoradiation to minimize patient inconvenience and discomfort. All patients will be asked to fill out patient-reported quality of life (QOL) questionnaires (UW-QOL and FACT-HN). All patients will have a clinical nutritional assessment by the study dietitian prior to initiating treatment or within the first week ($\leq$5 treatment sessions) of treatment.

All patients will participate in the Swallow Preservation Program in the Speech Pathology Clinic. They will begin participation within one week ($\geq$5 treatment sessions) of initiating chemoradiation. They must pass the Clinical Swallow Evaluation (CSE) that occurs during the Swallow Preservation Program initial assessment or deemed to have a functional oropharyngeal swallow with a Modified Barium Swallow Study (MBSS) if unable to pass the CSE for continuation in the study. Teaching to minimize dysphagia will be administered, and evaluation for evidence of aspiration will also be done throughout participation in the Swallow Preservation Program.

5.4 Weekly On-Treatment Management & Nutritional Supplementation

Weekly during treatment, patients will have their body weight, body mass index, and nutritional status (oral intake) assessed by the dietitian. If the dietitian is unavailable, a member of the clinical team will evaluate body weight and body mass changes, as well as take estimates of oral intake. The body weight at the first on-treatment visit during chemoradiation will be compared to the body weight taken at the time of enrollment. If a patient has gained weight, this new body weight will be used as the baseline weight from which weight loss will be determined. However, if weight loss occurs from enrollment, the baseline body weight will remain the body weight at the time of enrollment. Patients will also have weekly assessment of physician-reported acute toxicities during weekly on-treatment visits with the treating radiation oncologist.

The radiation oncologist, in conjunction with the dietitian, will determine whether the patient has lost 5% of body weight from baseline, indicating the need to initiate oral nutritional replacement with Soylent™. The dietitian will record weekly oral intakes for all patients, including the use of oral nutritional replacement. Patients will be monitored through the Swallow Preservation Program for evidence of aspiration.

Once 5% body weight loss is seen, patients will be given a small supply of Soylent™ from the clinic while awaiting their home order to be filled. In order to receive this delivery, patients will be

given a voucher for online redemption, with which they will sign up for delivery from Soylent™. Soylent™ will not ask for any information in addition to what is standardly required for delivery service. Please see Appendix for a screenshot of this sign-up process.

Compliance with oral nutrition replacement is defined as continuing at least 50% of recommended supplementation with Soylent™ as recommended by dietitian. If a patient stops all use of Soylent™ during treatment without resumption (and dietitian recommendation is for continued use of oral nutritional supplementation), this will be considered non-compliance. If a patient is maintaining weight on regular nutrition without supplementation, or proceeds with G-tube placement, stopping Soylent™ supplementation will not be considered non-compliance. If a patient is found to have evidence of aspiration in the Swallow Preservation Program, discontinuation of Soylent™ replacement will not be considered non-compliance. If a patient refuses to resume Soylent™ (and is considered non-compliant), alternative nutritional recommendations will be given to the patient from the dietitian and treating radiation oncology team. Using other nutritional supplementation in addition to Soylent™ (including regular food, other commercially available shakes, etc.) will not be considered non-compliance. However, it will be noted in the nutritional record that the patient is using means in addition to Soylent™ for nutrition.

Early G-tube insertion if it is in the best medical judgement by the treating physicians may be done prior to the 10% weight loss. If a patient loses >10% of weight and is determined to necessitate a G-tube due to poor oral nutritional intake by the treating radiation oncologist, in conjunction with the dietitian assessment, a therapeutic G-tube will be recommended for the patient. Therapeutic G-tube placement will be performed by the interventional radiology or gastroenterology services at the study site. Upon completion of concurrent chemoradiation (last day of treatment), a second set of serum/cheek swab samples will be collected along with repeat UW-QOL and FACT-HN questionnaires. These labs will again be drawn along with necessary on-treatment labs for chemoradiation to minimize patient inconvenience and discomfort.

5.5 Patient Follow-Up after Treatment

Patients will be followed clinically after treatment per standard of care, including routine evaluation of patient-reported QOL metrics (UW-QOL and FACT-HN questionnaires), physician-reported acute and late toxicities, body weight, and clinical/radiographic evaluations for local/locoregional recurrence, and distant metastases. A patient death, and its cause, will be recorded by the treating radiation oncologist in the study records if this occurs. Patients will continue routine follow-up visits in radiation oncology at approximately 1, 3, 6, 12, 16, 18, and 24 months as is routine at our institution for patients with locally advanced head and neck cancer surveillance. When practical and in the best interest of the subject, follow-up visits may occur via telemedicine. The UW-QOL and FACT-HN questionnaires may be administered electronically or by telephone. Serum (50 mls)/cheek swab samples will be taken only at the conclusion of chemoradiation (in the last week of treatment, as noted above) and at the 3-month follow-up visit. The 3-month follow-up visit labs will attempt to be drawn with necessary labs, if needed. Otherwise, this will be a separate blood draw. All serum and cheek swab sampling done for basic science research (i.e., those described in this section) will occur only when feasible

6.0 PHARMACEUTICAL INFORMATION

6.1 Investigational Agent or Device

Not applicable

6.2 Availability
Not applicable

6.3 Agent Ordering
Not applicable
6.4 Agent Accountability
Not applicable

7.0 ADVERSE EVENTS: LIST AND REPORTING REQUIREMENTS

Toxicity assessment will be performed using the National Cancer Institute Common Terminology Criteria for Adverse Events (CTCAE) version 4.0 for early (≤ 3 months), and late (> 3 months) radiation toxicity. This study's intervention is the addition of Soylent™ oral nutritional replacement triggered by 5% weight loss from baseline to standard chemoradiation for locally advanced head and neck cancer. The interaction of this nutritional supplement with radiation and chemotherapy toxicities is unknown but no specific toxicities are predicted as compared to patients undergoing standard chemoradiation treatment with standard supportive care measures.

7.1 Soylent™ Oral Nutrition Supplement-Related Toxicities
As Soylent™ is a commercially-available nutritional supplement/meal replacement agent with no known side effects, we do not specifically anticipate side effects from its use. Since it needs to be swallowed, potential adverse events in the mouth or throat are possibilities, including worsened oral mucositis, dysgeusia, dysphagia, or xerostomia which are known radiation and/or chemotherapy-related toxicities for chemoradiation to the head and neck. Since Soylent™ may also impact the gastrointestinal system as a food, increased esophagitis, nausea/vomiting, dyspepsia, constipation, diarrhea, flatulence, and abdominal pain are possibilities. A food allergic reaction, including anaphylaxis, is a possibility. Possible additional toxicities may include exacerbation of any other radiation-related or chemotherapy-related toxicity due to an unknown mechanism.

8.0 STUDY CALENDAR

**Study procedures**	**Screening**	**Pre-CRT**	**CRT**	**Post treatment visit (months following chemoradiation)**							
				**1**	**3**	**6**	**12**	**16**	**18**	**24**	**ET**
IMRT			X								
Chemotherapy			X								
Informed Consent	X										
Demographics	X										
Medical History	X										
Initial Consultation with Radiation Oncology	X										
Initial Consultation with Medical Oncology		X									
Initial Consultation with the dietitian		***X									
Urine Pregnancy Testing/Pregnancy Waiver as Clinically Indicated*	X										
Radiation Treatment Planning: CT, PET-CT, and/or MRI-based simulation		X									
Swallow Preservation Program		***X	X**	X**							
Weekly On-Treatment Visit with Radiation Oncologist			X								
Weekly On-Treatment Visit with the dietitian			X								
Body Weight Measurement/BMI Calculation	X	X	X	X	X	X	X	X	X	X	X
Research Blood and Cheek Swab Sampling	X[a]	☸X[a]	X[a]		X[a]						
Soylent™ Oral Nutrition Replacement			X								
Follow-up visit				X	X	X	X	X	X	X	X
QOL Questionnaires (UW-QOL and FACT-HN)		X		X	X	X		X	X		X

***Pregnancy testing/pregnancy waiver is given in our department as clinically indicated prior to the administration of radiation therapy (pre- or peri-menopausal woman under the age of 55 years)**

**Patients will attend swallow preservation program sessions as determined by the Speech Pathology Clinic team. This

may include sessions during or following chemoradiation.
CRT= chemoradiation
*** Must occur within one week ($\geq$ 5) treatment sessions of initiated chemo-radiation.
Routine follow-up visits every 3 months for the first year and every 4 months in the second year.
☸ Pre-treatment (Baseline) labs, if not already collected at consent.
[a] Collect the final translational lab draw/ Swab collection at the last week of end of RT treatment. All saliva and blood collection done for basic science research purposes will occur whenever feasible.

9.0 DATA REPORTING/REGULATORY CONSIDERATIONS

9.1 Data Management

The principal investigator and research coordinator will be responsible for the database records of patient data. The data will be kept on the research coordinator's computer under password protection. A chart with all the relevant research patient information will be maintained for each patient by the research coordinator.

9.2 Confidentiality

Study data will be maintained in password protected computer files. Only research personnel will have access to this information. When possible, identifiers will be removed. Only research personnel and investigators will keep the study data along with identifiers together in the same database under password protection accessible.

10. STATISTICAL CONSIDERATIONS

10.1 Study Endpoints

10.1.1 Sample Size and Power Consideration

This is a single arm, phase II prospective study to evaluate Soylent™ oral nutritional supplementation for compliance rate and potential impact on therapeutic G-tube rate, nutrition, quality of life, and clinical outcomes with its use during and immediately following chemoradiation in the definitive or adjuvant setting for head and neck cancer. The primary endpoints are compliance rate of Soylent™ oral nutrition supplementation and therapeutic G-tube rate. We plan to enroll 60 patients.

One primary endpoint is to ensure compliance with Soylent™ nutritional supplementation in patients undergoing concurrent chemoradiation. For feasibility in using this supplement for head and neck cancer patients, a goal of more than 50% of patients should continue to use this supplement (to at least 50% of recommended quantities) throughout treatment once initiated.

A therapeutic G-tube placement rate 33% lower than institutional historical control data is the second primary endpoint for this study. Our historical control data notes a 32% G-tube rate for chemoradiation patients, so the goal endpoint is 20%. If, conversely, a 25% higher rate of therapeutic G-tube placement occurs (equating to a 40% G-tube placement rate), this should trigger study stoppage. A sample size of 60 patients achieves 80% power to detect a 33% reduction in G-tube placement rate when comparing to historical control patients (i.e. 20% vs 32%), using a one-sided one-sample exact Binomial test, at a 0.10 significance level.

10.2 Planned Methods of Analysis

10.2.1. Analysis of Primary Endpoints

For the analysis of compliance, we will calculate the compliance rate and the corresponding 95% exact confidence interval. The G-tube placement rate will be analyzed using one-sample exact Binomial test. The 95% exact confidence interval (CI) will be provided.

10.2.2. Analysis of Secondary Endpoints

Weight loss and body mass index (BMI) will be collected at each visit and summary descriptive statistics will be calculated at each time points. Repeated measure ANOVA will be used to explore if there are any change in these parameters over time. Treatment breaks will be recorded in days per study patient and summarized. G-tube placement timing will be recorded for patients receiving G-tubes, and the duration of G-tube dependence will be recorded in days per study patient and summarized.

Serum HPV DNA, pre-albumin, albumin, C-reactive protein (CRP), interleukin-6 (IL-6), tumor necrosis factor (TNF), Vitamin A, Vitamin C, Vitamin D, tryptophan, kynurenine, leptin, and adiponectin, complete metabolic panel, and complete blood count and radiosensitivity biomarker panel will be summarized by descriptive statistics by visit time points, and these statistics will be plotted graphically.

Patient-reported QOL according to the University of Washington (UW-QOL) and Functional Assessment of Cancer Therapy-Head & Neck (FACT-HN) questionnaires will be summarized by descriptive statistics by visit time points.

The Kaplan-Meier method will be used to estimate time-to-event endpoints such as local/locoregional control, distant metastasis-free survival and overall survival. Summaries of the number and percentage of patients who have metastasized cancer/died, are still in survival follow-up, are lost to follow-up and have withdrawn consent will be provided along with median distant metastasis-free survival/overall survival.

As exploratory analyses, we will compare these secondary outcome measurements from our study with the control group or previously conducted studies available in the literature to evaluate the potential benefits of adding Soylent™ oral nutrition meal replacement in patients with head & neck cancer undergoing definitive or adjuvant chemoradiotherapy.

10.2.3 Control Group Comparison

An institutional historical control patient cohort, which was used to determine the historical G-tube rate of 32%, will be used for comparative analyses with our study cohort.

10.2.4 Safety Analysis

Toxicity will be graded using the NCI Common Toxicity Criteria for Adverse Events (CTCAE) version 4.0. AEs and SAEs will be reported using a CTCAE v4.0 terminology and severity. Given that this is a food without any known properties to interact adversely with chemoradiation for head and neck cancer, we anticipate no specific toxicities which should trigger stopping the study. However, toxicity will be monitored and a 10% rate of grade 3 or higher toxicity attributable to Soylent™ will trigger study stoppage.

10.3 Patient Accrual and Study Duration

It is expected that it will take approximately 1 year to complete the study accrual. Study-related data will be stored for 5 years after termination of the study when accrual is no longer taking place and all patients have discontinued follow-up procedures.

10.4 Interim Analysis

There will be one planned interim analysis for G-tube rate, when data for the primary outcome measure are available for 50% of the original sample size (30 of 60 patients planned for enrollment). We will predict the G-tube rate (and confidence interval) at the end of the study condition based on the data observed thus far and assume a continuation of this trend at the end of the study.

10.5 Criteria for removal from study

10.5.1 The patient withdraws.

10.5.2 The investigator may withdraw a patient from the study for one or more of the following reasons: failure of the patient to follow instructions of the protocol study staff, the investigator decides that continuing participation could be harmful to the patient, the patient is not tolerating the Soylent™ meal replacement, the patient needs treatment not allowed in the study, the study is cancelled, other administrative reasons, or unanticipated circumstances.

10.5.3 The patients fail Swallow Preservation Program Clinical Swallow Evaluation (CSE) and Modified Barium Swallow (MBSS).

1. Appendix.

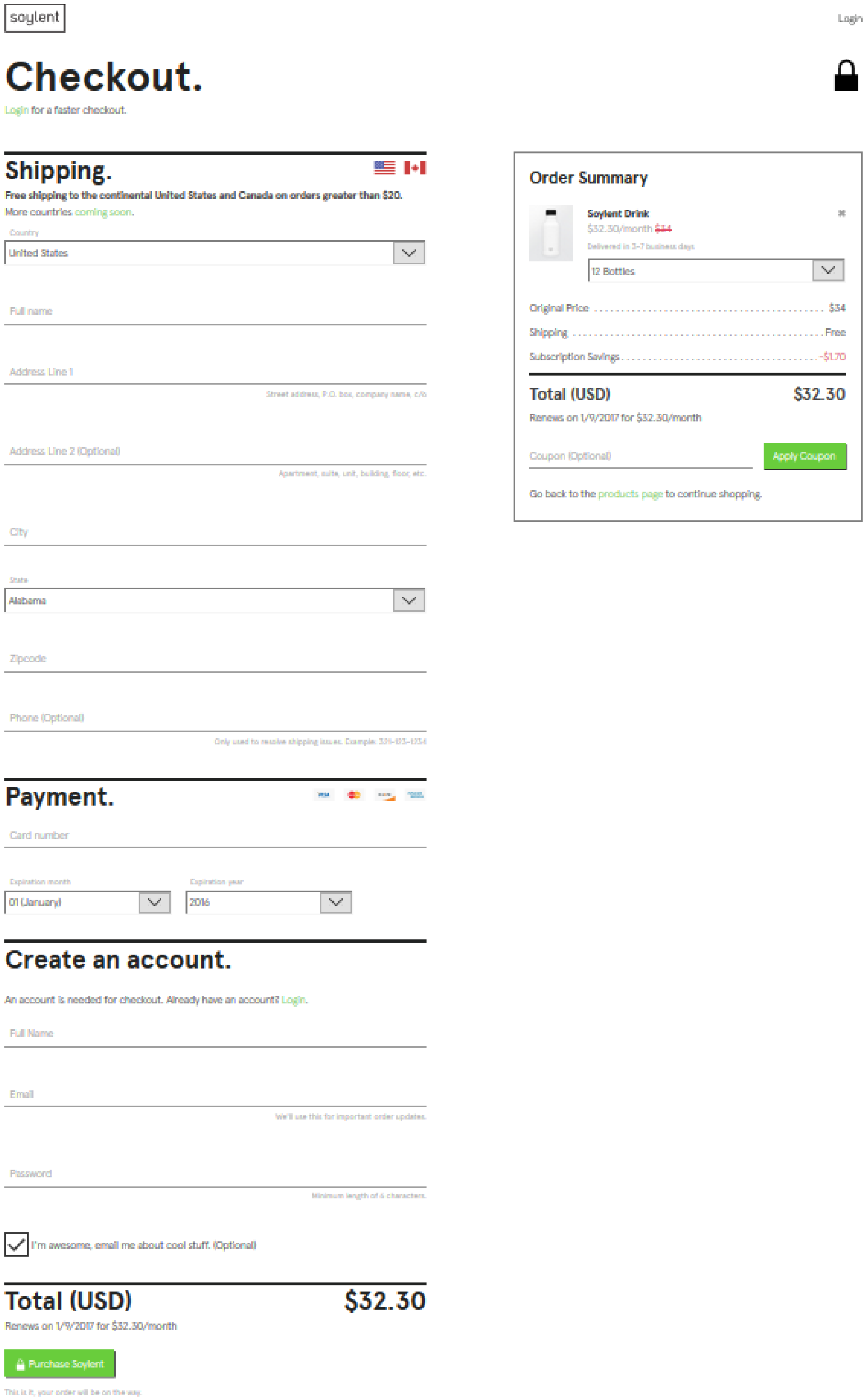

[redacted]

Soylent intake of product (Compliance)

Sunday<br>Date:	Monday<br>Date:	Tuesday<br>Date:	Wednesday<br>Date:	Thursday<br>Date:	Friday<br>Date:	Saturday<br>Date:
❒ Breakfast<br>❒ _______<br>❒ Lunch<br>❒ _______<br>❒ Dinner<br>❒ _______	❒ Breakfast<br>❒ _______<br>❒ Lunch<br>❒ _______<br>❒ Dinner<br>❒ _______	❒ Breakfast<br>❒ _______<br>❒ Lunch<br>❒ _______<br>❒ Dinner<br>❒ _______	❒ Breakfast<br>❒ _______<br>❒ Lunch<br>❒ _______<br>❒ Dinner<br>❒ _______	❒ Breakfast<br>❒ _______<br>❒ Lunch<br>❒ _______<br>❒ Dinner<br>❒ _______	❒ Breakfast<br>❒ _______<br>❒ Lunch<br>❒ _______<br>❒ Dinner<br>❒ _______	❒ Breakfast<br>❒ _______<br>❒ Lunch<br>❒ _______<br>❒ Dinner<br>❒ _______
Comments	*Comments*	*Comments*	*Comments*	*Comments*	*Comments*	*Comments*
Sunday<br>Date:	Monday<br>Date:	Tuesday<br>Date:	Wednesday<br>Date:	Thursday<br>Date:	Friday<br>Date:	Saturday<br>Date:
❒ Breakfast<br>❒ _______<br>❒ Lunch<br>❒ _______<br>❒ Dinner<br>❒ _______	❒ Breakfast<br>❒ _______<br>❒ Lunch<br>❒ _______<br>❒ Dinner<br>❒ _______	❒ Breakfast<br>❒ _______<br>❒ Lunch<br>❒ _______<br>❒ Dinner<br>❒ _______	❒ Breakfast<br>❒ _______<br>❒ Lunch<br>❒ _______<br>❒ Dinner<br>❒ _______	❒ Breakfast<br>❒ _______<br>❒ Lunch<br>❒ _______<br>❒ Dinner<br>❒ _______	❒ Breakfast<br>❒ _______<br>❒ Lunch<br>❒ _______<br>❒ Dinner<br>❒ _______	❒ Breakfast<br>❒ _______<br>❒ Lunch<br>❒ _______<br>❒ Dinner<br>❒ _______
Comments	*Comments*	*Comments*	*Comments*	*Comments*	*Comments*	*Comments*
Sunday<br>Date:	Monday<br>Date:	Tuesday<br>Date:	Wednesday<br>Date:	Thursday<br>Date:	Friday<br>Date:	Saturday<br>Date:
❒ Breakfast<br>❒ _______<br>❒ Lunch<br>❒ _______<br>❒ Dinner<br>❒ _______	❒ Breakfast<br>❒ _______<br>❒ Lunch<br>❒ _______<br>❒ Dinner<br>❒ _______	❒ Breakfast<br>❒ _______<br>❒ Lunch<br>❒ _______<br>❒ Dinner<br>❒ _______	❒ Breakfast<br>❒ _______<br>❒ Lunch<br>❒ _______<br>❒ Dinner<br>❒ _______	❒ Breakfast<br>❒ _______<br>❒ Lunch<br>❒ _______<br>❒ Dinner<br>❒ _______	❒ Breakfast<br>❒ _______<br>❒ Lunch<br>❒ _______<br>❒ Dinner<br>❒ _______	❒ Breakfast<br>❒ _______<br>❒ Lunch<br>❒ _______<br>❒ Dinner<br>❒ _______
Comments	*Comments*	*Comments*	*Comments*	*Comments*	*Comments*	*Comments*
Sunday<br>Date:	Monday<br>Date:	Tuesday<br>Date:	Wednesday<br>Date:	Thursday<br>Date:	Friday<br>Date:	Saturday<br>Date:
❒ Breakfast<br>❒ _______<br>❒ Lunch<br>❒ _______<br>❒ Dinner<br>❒ _______	❒ Breakfast<br>❒ _______<br>❒ Lunch<br>❒ _______<br>❒ Dinner<br>❒ _______	❒ Breakfast<br>❒ _______<br>❒ Lunch<br>❒ _______<br>❒ Dinner<br>❒ _______	❒ Breakfast<br>❒ _______<br>❒ Lunch<br>❒ _______<br>❒ Dinner<br>❒ _______	❒ Breakfast<br>❒ _______<br>❒ Lunch<br>❒ _______<br>❒ Dinner<br>❒ _______	❒ Breakfast<br>❒ _______<br>❒ Lunch<br>❒ _______<br>❒ Dinner<br>❒ _______	❒ Breakfast<br>❒ _______<br>❒ Lunch<br>❒ _______<br>❒ Dinner<br>❒ _______
Comments	*Comments*	*Comments*	*Comments*	*Comments*	*Comments*	*Comments*

Additional comments:

12. REFERENCES

1. van Bokhorst-de van der S, van Leeuwen PA, Kuik DJ, et al: The impact of nutritional status on the prognoses of patients with advanced head and neck cancer. Cancer 86:519-27, 1999
2. Rosenthal DI: Consequences of mucositis-induced treatment breaks and dose reductions on head and neck cancer treatment outcomes. J Support Oncol 5:23-31, 2007
3. Suntharalingam M, Haas ML, Van Echo DA, et al: Predictors of response and survival after concurrent chemotherapy and radiation for locally advanced squamous cell carcinomas of the head and neck. Cancer 91:548-54, 2001
4. Capuano G, Grosso A, Gentile PC, et al: Influence of weight loss on outcomes in patients with head and neck cancer undergoing concomitant chemoradiotherapy. Head Neck 30:503-8, 2008
5. Liu SA, Tsai WC, Wong YK, et al: Nutritional factors and survival of patients with oral cancer. Head Neck 28:998-1007, 2006
6. Brookes GB: Nutritional status--a prognostic indicator in head and neck cancer. Otolaryngol Head Neck Surg 93:69-74, 1985
7. Langius JA, Zandbergen MC, Eerenstein SE, et al: Effect of nutritional interventions on nutritional status, quality of life and mortality in patients with head and neck cancer receiving (chemo)radiotherapy: a systematic review. Clin Nutr 32:671-8, 2013
8. Ravasco P: Aspects of taste and compliance in patients with cancer. Eur J Oncol Nurs 9 Suppl 2:S84-91, 2005
9. Valentini V, Marazzi F, Bossola M, et al: Nutritional counselling and oral nutritional supplements in head and neck cancer patients undergoing chemoradiotherapy. J Hum Nutr Diet 25:201-8, 2012
10. Ravasco P, Monteiro-Grillo I, Marques Vidal P, et al: Impact of nutrition on outcome: a prospective randomized controlled trial in patients with head and neck cancer undergoing radiotherapy. Head Neck 27:659-68, 2005
11. van den Berg MG, Rasmussen-Conrad EL, Wei KH, et al: Comparison of the effect of individual dietary counselling and of standard nutritional care on weight loss in patients with head and neck cancer undergoing radiotherapy. Br J Nutr 104:872-7, 2010
12. Oozeer NB, Corsar K, Glore RJ, et al: The impact of enteral feeding route on patient-reported long term swallowing outcome after chemoradiation for head and neck cancer. Oral Oncol 47:980-3, 2011
13. Corry J, Poon W, McPhee N, et al: Prospective study of percutaneous endoscopic gastrostomy tubes versus nasogastric tubes for enteral feeding in patients with head and neck cancer undergoing (chemo)radiation. Head Neck 31:867-76, 2009
14. Chen AM, Li BQ, Lau DH, et al: Evaluating the role of prophylactic gastrostomy tube placement prior to definitive chemoradiotherapy for head and neck cancer. Int J Radiat Oncol Biol Phys 78:1026-32, 2010
15. Bossola M: Nutritional interventions in head and neck cancer patients undergoing chemoradiotherapy: a narrative review. Nutrients 7:265-76, 2015
16. Lambrecht M, Nevens D, Nuyts S: Intensity-modulated radiotherapy vs. parotid-sparing 3D conformal radiotherapy. Effect on outcome and toxicity in locally advanced head and neck cancer. Strahlenther Onkol 189:223-9, 2013
17. Vainshtein JM, Moon DH, Feng FY, et al: Long-term quality of life after swallowing and salivary-sparing chemo-intensity modulated radiation therapy in survivors of human papillomavirus-related oropharyngeal cancer. Int J Radiat Oncol Biol Phys 91:925-33, 2015

18. Al-Mamgani A, van Rooij P, Verduijn GM, et al: The impact of treatment modality and radiation technique on outcomes and toxicity of patients with locally advanced oropharyngeal cancer. Laryngoscope 123:386-93, 2013

19. Setton J, Lee NY, Riaz N, et al: A multi-institution pooled analysis of gastrostomy tube dependence in patients with oropharyngeal cancer treated with definitive intensity-modulated radiotherapy. Cancer 121:294-301, 2015

20. Nayel H, el-Ghoneimy E, el-Haddad S: Impact of nutritional supplementation on treatment delay and morbidity in patients with head and neck tumors treated with irradiation. Nutrition 8:13-8, 1992

21. Arnold C, Richter MP: The effect of oral nutritional supplements on head and neck cancer. Int J Radiat Oncol Biol Phys 16:1595-9, 1989

22. Bauer J, Capra S, Battistutta D, et al: Compliance with nutrition prescription improves outcomes in patients with unresectable pancreatic cancer. Clin Nutr 24:998-1004, 2005

23. Imamura H, Nishikawa K, Kishi K, et al: Effects of an Oral Elemental Nutritional Supplement on Post-gastrectomy Body Weight Loss in Gastric Cancer Patients: A Randomized Controlled Clinical Trial. Ann Surg Oncol 23:2928-35, 2016

24. Rutter CE, Yovino S, Taylor R, et al: Impact of early percutaneous endoscopic gastrostomy tube placement on nutritional status and hospitalization in patients with head and neck cancer receiving definitive chemoradiation therapy. Head Neck 33:1441-7, 2011

25. Jang JW, Parambi RJ, McBride SM, et al: Clinical factors predicting for prolonged enteral supplementation in patients with oropharyngeal cancer treated with chemoradiation. Oral Oncol 49:438-42, 2013

26. Li G, Gao J, Liu ZG, et al: Influence of pretreatment ideal body weight percentile and albumin on prognosis of nasopharyngeal carcinoma: Long-term outcomes of 512 patients from a single institution. Head Neck 36:660-6, 2014

27. Unal D, Orhan O, Eroglu C, et al: Prealbumin is a more sensitive marker than albumin to assess the nutritional status in patients undergoing radiotherapy for head and neck cancer. Contemp Oncol (Pozn) 17:276-80, 2013

28. Lin A, Jabbari S, Worden FP, et al: Metabolic abnormalities associated with weight loss during chemoirradiation of head-and-neck cancer. Int J Radiat Oncol Biol Phys 63:1413-8, 2005

29. Silver HJ, Dietrich MS, Murphy BA: Changes in body mass, energy balance, physical function, and inflammatory state in patients with locally advanced head and neck cancer treated with concurrent chemoradiation after low-dose induction chemotherapy. Head Neck 29:893-900, 2007

30. Chung MK, Kim DH, Ahn YC, et al: Randomized Trial of Vitamin C/E Complex for Prevention of Radiation-Induced Xerostomia in Patients with Head and Neck Cancer. Otolaryngol Head Neck Surg, 2016

31. Meyer F, Liu G, Douville P, et al: Dietary vitamin D intake and serum 25-hydroxyvitamin D level in relation to disease outcomes in head and neck cancer patients. Int J Cancer 128:1741-6, 2011

32. de Luis DA, Izaola O, Cuellar L, et al: A randomized clinical trial with two doses of a omega 3 fatty acids oral and arginine enhanced formula in clinical and biochemical parameters of head and neck cancer ambulatory patients. Eur Rev Med Pharmacol Sci 17:1090-4, 2013

33. de Luis DA, Izaola O, Cuellar L, et al: Clinical and biochemical outcomes after a randomized trial with a high dose of enteral arginine formula in postsurgical head and neck cancer patients. Eur J Clin Nutr 61:200-4, 2007

34. de Luis DA, Izaola O, Cuellar L, et al: Randomized clinical trial with an enteral arginine-enhanced formula in early postsurgical head and neck cancer patients. Eur J Clin Nutr 58:1505-8, 2004

35. Yeh KY, Wang HM, Chang JW, et al: Omega-3 fatty acid-, micronutrient-, and probiotic-enriched nutrition helps body weight stabilization in head and neck cancer cachexia. Oral Surg Oral Med Oral Pathol Oral Radiol 116:41-8, 2013

36. Ang KK, Harris J, Wheeler R, et al: Human papillomavirus and survival of patients with oropharyngeal cancer. N Engl J Med 363:24-35, 2010

37. de Jong MC, Pramana J, Knegjens JL, et al: HPV and high-risk gene expression profiles predict response to chemoradiotherapy in head and neck cancer, independent of clinical factors. Radiother Oncol 95:365-70, 2010

38. Kumar B, Cordell KG, Lee JS, et al: Response to therapy and outcomes in oropharyngeal cancer are associated with biomarkers including human papillomavirus, epidermal growth factor receptor, gender, and smoking. Int J Radiat Oncol Biol Phys 69:S109-11, 2007

39. Wang Y, Springer S, Mulvey CL, et al: Detection of somatic mutations and HPV in the saliva and plasma of patients with head and neck squamous cell carcinomas. Sci Transl Med 7:293ra104, 2015
